# Subjective social status is associated with happiness but not weight status or psychological distress: An analysis of three prospective birth cohorts from low- and middle-income countries

**DOI:** 10.1016/j.wss.2022.100115

**Published:** 2022

**Authors:** Jithin Sam Varghese, Rachel Waford Hall, Linda S Adair, Shivani A Patel, Reynaldo Martorell, Delia E. Belleza, Maria F Kroker-Lobos, Nanette R. Lee, Lukhanyo H. Nyati, Manuel Ramirez-Zea, Linda M Richter, Aryeh D. Stein

**Affiliations:** a Nutrition and Health Sciences Program, Laney Graduate School, Emory University, Atlanta, GA, USA; b Hubert Department of Global Health, Rollins School of Public Health, Emory University, Atlanta, GA, USA; c Department of Nutrition, Gillings School of Global Public Health, University of North Carolina at Chapel Hill, Chapel Hill, NC, USA; d Department of Psychology, University of San Carlos, Cebu City, Philippines; e INCAP Research Center for the Prevention of Chronic Diseases (CIIPEC), Institute of Nutrition of Central America and Panama (INCAP), Guatemala City, Guatemala; f USC-Office of Population Studies Foundation, Inc, University of San Carlos, Cebu City, Philippines; g SAMRC Developmental Pathways for Health Research Unit, University of the Witwatersrand, Johannesburg, South Africa; h DSI-NRF Centre of Excellence in Human Development, University of the Witwatersrand, Johannesburg, South Africa

**Keywords:** Subjective social status, Perceived community respect, Perceived economic status, Socioeconomic status, Body mass index, Happiness, BMI, Body Mass Index, LMIC, Low- and middle-income country, PCA, Principal Component Analysis, SEP, Socio-economic Position, SHS, Subjective Happiness Scale, SRQ-20, World Health Organization Self-Reporting Questionnaire-20, SSS, Subjective Social Status

## Abstract

**Background:**

Subjective social status (SSS, perception of social position relative to a frame of reference) has been associated with physical, mental and socio-emotional wellbeing. However, these associations may be susceptible to unmeasured confounding by life course objective socio-economic position (SEP; such as wealth, education and employment) and life satisfaction.

**Purpose:**

To estimate the association of position on ladders of perceived community respect and perceived economic status with weight, distress and wellbeing, independent of objective SEP in cohorts from three low and middle-income countries.

**Methods:**

We used data from birth cohorts in Guatemala (n = 1258), Philippines (n = 1323) and South Africa (n = 1393). We estimated the association of perceived community respect and perceived economic status with body mass index (kg/m^2^), the World Health Organization's Self-Reported Questionnaire-20 (SRQ-20) for psychological distress, and Lyubomirsky's Subjective Happiness Scale. We estimated these associations using robust linear regression models adjusting for indicators of life course objective SEP, early life characteristics, adult covariates, and life satisfaction.

**Results:**

Participants in South Africa (age 27-28y) rated themselves higher on average for both the respect (7 vs 5 in Guatemala and 6 in Philippines) and economic (5 vs 3 in Guatemala and 4 in Philippines) ladder measures. Position on neither community respect nor economic ladders were associated with BMI or psychological distress. Higher position on community respect (Guatemala: 0.03, 95%CI: 0.01, 0.04; Philippines: 0.03, 95% CI: 0.02, 0.05; South Africa: 0.07, 95%CI: 0.04, 0.09) and economic (Guatemala: 0.02, 95%CI: 0, 0.04; Philippines: 0.04, 95%CI: 0.02, 0.07; South Africa: 0.07, 95%CI: 0.04, 0.10) ladders were associated with greater happiness.

**Conclusions:**

Subjective social status showed small but consistent associations with happiness in birth cohorts independent of life-course SEP.

## Highlights

• Previous studies of association of subjective social status with health are susceptible to unmeasured confounding by life course socio-economic position and life satisfaction.
• Subjective social status was not associated with body mass index or psychological distress in three birth cohorts from Guatemala, Philippines and South Africa.
• Higher position on community respect and economic ladders were associated with greater happiness.

## 1 Introduction

Subjective social status (SSS) is one's own evaluation of their socio-economic position (SEP) relative to others (Adler et al., 2000). SEP is a function of material (e.g. wealth), human (e.g. schooling) and social (e.g. networks) capital (Oakes and Rossi, 2003). Higher SSS has been found to be associated with lower rates of depression, cardiovascular disease and all-cause mortality, independent of objective SEP measures such as wealth, education and employment status (Singh-Manoux et al., 2003; Euteneuer, 2014; Bradshaw et al., 2017; Schubert et al., 2016). Given the self-reported nature of SSS and its associations with health measures such as self-rated health and psychological distress, some possible critiques included correlated measurement error and confounding by temporary mood (Kraus et al., 2013; Keogh et al., 2020). However, experimental studies of allocation into upward or downward comparison standards for SSS showed that such associations are robust to confounding by temporary mood (Kraus et al., 2013; Jackson et al., 2011).

**Fig. 1:**
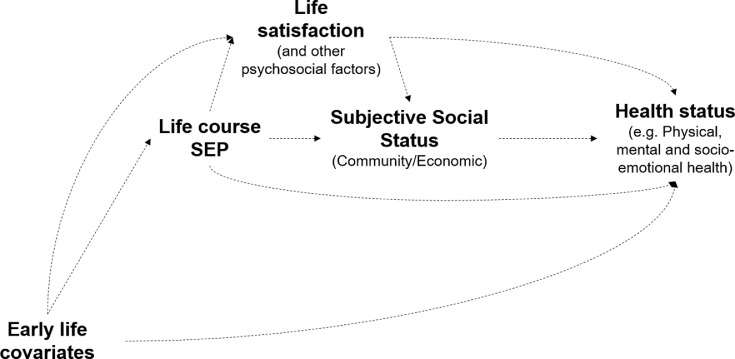
Framework for association of subjective social status with health and wellbeing. SEP: Socio-economic position; Life satisfaction and subjective social status may be determined by prior health status.

We present a conceptual framework of how SSS is associated with health in Fig. 1. SEP over the life course is associated with life satisfaction (evaluative wellbeing) (Killingsworth, 2021; Niedzwiedz et al., 2014). SSS is considered to be a ‘cognitive average’ of objective SEP relative to others in their community (Singh-Manoux et al., 2003). SSS may also be influenced by personality traits and factors such as locus of control and life satisfaction (Hoebel and Lampert, 2020). Previous research exploring mediation of the association of SSS and health-related stress responses by general life stressors showed null findings and suggested that alternate mediating pathways directly related to social status related stressors (such as financial stress and dominance) are understudied (Cundiff et al., 2020).

**Fig. 2:**
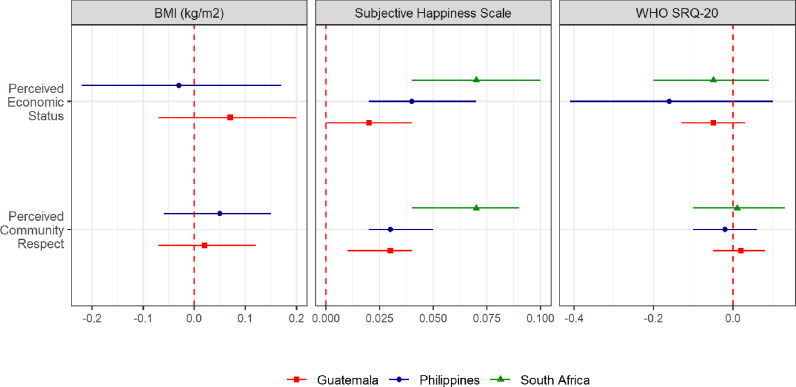
Associations of SSS with health and wellbeing outcomes with adjustment for life course SEP measures by cohort. Associations were estimated from linear regressions. Models (Model 3C, Model 3E) were fit separately for perceived community respect and perceived economic status) adjusted for socio-economic position (wealth in childhood and adulthood, maternal and attained schooling, formal employment), early life covariates and adult covariates (including life satisfaction, marital status and children – yes/no).

Observational studies from high-income settings suggest that associations of SSS and health vary by outcome, race, sex and country (Subramanyam et al., 2012; Allen et al., 2014; Shaked et al., 2016). The scarce evidence on heterogeneous associations from cross-sectional surveys in low- and middle-income countries (LMICs) is consistent with available evidence from high-income settings (Sasaki et al., 2021; Prag et al., 2016). However, research on SSS may be susceptible to epidemiological biases further reducing generalizability – specifically correlated measurement error from self-reported instruments and unmeasured confounders such as life course objective SEP, life satisfaction and personality traits (e.g. self-esteem) (Hoebel and Lampert, 2020). Unbiased estimates for association of SSS with health are important to design appropriate interventions.

Using birth cohorts from three LMICs, we explore our a priori hypothesis (derived from our conceptual framework in Fig. 1) that subjective social status was associated with health beyond life course wealth, own and parental schooling and life satisfaction. Consistent associations across different LMIC settings in cohorts born across a range of birth years, with varying environmental exposures, and confounding structures would suggest generalizable results (Richmond et al., 2014). Our objective is to study the association of SSS on multiple dimensions of health, i.e. physical, mental and socio-emotional well-being, among study populations in three countries, independent of objective SEP (World Health Organization, 1948).

## 2 Methods

### 2.1 Study population

We use life-course data from three birth cohorts that are part of the Consortium of Health Oriented Research in Transitioning Societies (COHORTS) collaborative (Richter et al., 2012). The cohorts are from countries in three different continents: Guatemala (INCAP Longitudinal Study), Philippines (Cebu Longitudinal Health and Nutrition Survey, born in 1983-84) and South Africa (Birth to Twenty plus Cohort, born in 1990) (Stein et al., 2008; Adair et al., 2011; Richter et al., 2007). The Guatemala cohort was followed from birth only for those born in the period 1969 to 1977. We obtained ethical approval for this secondary analysis from Emory University IRB (Protocol 95960).

### 2.2 Data collection and variable specification

#### 2.2.1 Subjective social status

Subjective Social Status was measured using the MacArthur Ladder in adulthood (2017-18; Guatemala: 37-55y, Philippines: 35-36y, South Africa: 27-28y) (Singh-Manoux et al., 2003). Participants were asked to visualize a ladder that represents their community. They were asked the following questions to assess (a) Perceived Community Respect (‘respect ladder’), and (b) Perceived Economic Status (‘economic ladder’) respectively relative to other people around them in their community or neighborhood, similar to other formulations (SPARQtools, 2022). The reference population for the ladder is not provided in the original formulation, and is considered to be the society in general (Adler et al., 2000).

(a) At the top of ladder are people who have the highest standing in the community. They are the most respected members of the community. At the bottom are the people who have the lowest standing in the community and who are least respected. At this time in your life, relative to other people in your community, what rung of the ladder do you think you stand on, from 1 (least respected) to 10 (most respected)?
(b) At the top of the ladder are people who are the richest, those who have the most money or wealth. At the bottom are the people who are the poorest, those who have the least money or wealth. At this time in your life, relative to other people in your community, what rung of the ladder do you think you stand on, from 1 (worst off) to 10 (best off)?

We included the words ‘and the most respectable jobs’ to the respect ladder after pilot testing for Guatemala only. Therefore, this measure may include components of economic standing, apart from respect in the community due to its secondary focus on employment. Though the ladder measures were not validated in these specific contexts, these measures are similar to those from previous studies in LMIC settings where the MacArthur ladder was used (Prag et al., 2016).

#### 2.2.2 Life course objective socio-economic position

Life course measures of objective SEP, namely wealth, schooling and employment, were available for all cohorts (Richter et al., 2012). Data on assets (self-reported possession) and housing characteristics were collected prospectively at each study wave for the three birth cohorts (Guatemala: 1967, 1975, 1987, 1996, 2002, 2015-18; Philippines: 1983-84, 1991-92, 1994-95, 1998-2000, 2002-03, 2005, 2009, 2018-19; South Africa: 1990-92, 1997-98, 2002-03, 2006-07, 2012-13, 2017-18). We developed cross-sectional asset indices, as measures of relative wealth, from contextually relevant sets of assets (such as television, car etc) and housing characteristics (house ownership, housing material etc). The number of assets varied for each study wave and cohort (Varghese et al., 2021). Although asset indices were available over the life course, we used the measures that were estimated in childhood (Guatemala: 1967 or 1975, Philippines: 1983-84, South Africa: 1990-92) and adulthood (Guatemala: 2015-18, Philippines: 2018-19, South Africa: 2017-18) (Varghese et al., 2021). We used the first component from a polychoric principal component analysis (PCA) of asset data and standardized it to unit variance to account for correlated nature of data and rank order households on wealth (Filmer and Pritchett, 2001). Asset-based indices are also correlated with non-food expenditures in societies where food expenditures constitute a minority of total expenditure and households do not experience transitory shocks to expenditure (Filmer and Scott, 2012). Schooling measures, collected at the most recent wave and at enrollment, for participants and their mothers, respectively, reflect final school attainment. Employment status in adulthood was classified into formal or informal/unemployed/not seeking work.

#### 2.2.3 Outcomes

We investigated three health and wellbeing outcomes: body mass index as a non-specific indicator of physical health, psychological distress as a measure of mental health and happiness as a measure of socio-emotional wellbeing (World Health Organization, 1948). Body mass index is a proxy for adiposity, a recognized marker of cardiometabolic disease risk.

Height and weight were measured in 2015-16 for Guatemala and in 2017-18 for Philippines using standardized protocols. We computed body mass index (BMI; kg/m^2^) as weight (kg) divided by square of height (m). Height and weight were not collected for the South African cohort at the time SSS data were collected. There was high prevalence of overweight or obese (BMI ≥ 25 kg/m^2^) in Guatemala (females: 78.2%, males: 62.9%) and Philippines (females: 47.5%, males: 44.9%), with low prevalence of underweight (BMI < 18.5 kg/m^2^; Guatemala: 9.8%, Philippines: 5.5%).

Psychological distress was measured using the WHO Self-Reported Questionnaire (SRQ-20), a 20-item instrument (per item; yes: 1, no: 0) which is widely used in low-resource settings as a screening tool for mental distress (Beusenberg and Orley, 1994). We summed the counts of psychological distress symptoms (range: 0 to 20). Subjective happiness was measured using the 4-item (per item; low: 1, high: 5) Lyubomirsky Subjective Happiness Scale (SHS) (Lyubomirsky and Lepper, 1999). We averaged the responses of SHS items to a range of 1 to 5. Both SRQ-20 and SHS were administered in 2017-18 for Guatemala and Philippines. For South Africa, SRQ-20 and one item from the SHS scale were asked in 2017-18.

#### 2.2.4 Early life and adult covariates

We adjusted for a common set of early life covariates (maternal age, maternal schooling, birth order and sex of participant) across all cohorts. Additionally, we adjusted for cohort-specific covariates (Guatemala: year and village of birth; Philippines: rural residence, South Africa: whether skin color was Black). We included adult covariates measured concurrently with the outcomes: adult life satisfaction (measured using NIH Toolbox Item Bank v2.0 – General Life Satisfaction), whether participants have children (yes/no), marital status (married/co-habiting versus not) and residence in adulthood (for Guatemala and Philippines, rural vs. urban) (Salsman et al., 2014). The South Africa cohort is comprised entirely of urban residents.

### 2.3 Statistical analysis

We carried out our analysis separately by cohort. The analytic sample was restricted to those participants who provided information on both measures of subjective social status and at least one outcome in adulthood (n; Guatemala: 1258, Philippines: 1323, South Africa: 1393). A flowchart for selection of the analytic sample is available in Supplementary Fig. 1. Since there were missing values for some covariates and outcomes, we used multiple imputation (10 datasets, 50 iterations, predictive mean matching) under missing at random assumptions. We included subjective social status (respect and economic ladders), life course objective SEP, auxiliary variables (early life covariates, adult covariates) and outcome variables (BMI, SRQ-20, SHS) in the imputation model. Outcome variables included in the imputation stage with auxiliary variables may provide more precise effect estimates and better coverage probability of 95% confidence intervals, without any other loss of performance (Kontopantelis et al., 2017; van Ginkel et al., 2020). We did not delete imputed outcomes from the analysis datasets.

We used linear regression with robust standard errors to estimate the association between each component of the SSS and our outcome variables separately even when normality and homoscedasticity assumptions of the residuals were violated. We accounted for clustering by maternal identifier in Guatemala and current barangay (neighborhood) of residence in Philippines using marginal models. We fit models without any covariates (Model 1) and sequentially adjusted (including all preceding variables) for SEP and life satisfaction (Model 2), early life and adult covariates (Model 3), and effect modification of association of SSS and outcomes by sex (Model 4a), attained schooling (Model 4b) or wealth in adulthood (Model 4c). We excluded women who were pregnant from analysis of BMI.

### 2.4 Sensitivity analysis

We performed three sensitivity analyses to assess the robustness of our findings (details in Supplementary Note 1). First, we repeated our analysis after adjusting for additional cross-sectional measures of life course wealth (in previous life stages) to estimate any residual confounding by SEP. Second, we used e-values to quantify the extent of unmeasured confounding of the SSS and outcome associations. The e-value for an exposure-outcome association is the minimum strength of association an unmeasured confounder should have with both the exposure (i.e. subjective social status) and outcome (BMI/SRQ-20/SHS) to nullify the observed association (VanderWeele and Ding, 2017). Third, we repeated our analysis after using inverse probability of censoring weights to account for non-participation (due to death or non-response) in adulthood (Seaman et al., 2012).

All analysis was carried out using R version 3.6.1.

## 3 Results

Descriptive characteristics of the analytic sample are provided in Table 1. Participants in South Africa (age 27-28y) rated themselves higher than those in the other two cohorts for both the respect (7 vs 5 in Guatemala and 6 in Philippines) and economic (5 vs 3 in Guatemala and 4 in Philippines) ladders.

**Table 1:** Early life and adult characteristics for analytic sample.

	**Guatemala (INCAP)** ***N* = 1258**		**Philippines (CLHNS)** ***N* = 1323**		**South Africa (Birth to Twenty plus)** ***N* = 1393**	
	N	Summary	N	Summary	N	Summary
***Subjective Social Status***						
Perceived Community Respect	1258	5 [3, 8]	1323	6 [5, 8]	1393	7 [5, 8]
Perceived Economic Status	1258	3 [1,5]	1323	4 [3,5]	1393	5 [4,5]
***Socio-economic position***						
Maternal schooling (years)	1221	1 [0,2]	1323	6 [5,9]	1290	9 [9,12]
Attained schooling (years)	1258	6 [2,6]	1323	11 [9,13]	1392	12 [11,12]
Formal employment	1251	48.8%	1323	35.0%	1365	43.6%
***Early life covariates***						
Maternal age (years)	1249	26 [21, 32]	1323	26 [22, 32]	1391	25 [21, 32]
Birth order	1247	4 [2, 4]	1323	3 [2, 4]	1393	2 [1, 3]
Male	1258	44.6%	1323	53.9%	1393	47.4%
Birth year	1258	1970 [1967, 1974]	1323	1983-84	1393	1990
Rural residence in childhood		-	1323	28.0%		
Black skin color		-		-	1393	88.4%
***Adult covariates***^a^						
Is married	1258	52.1%	1323	46.5%	1354	10.8%
Have children	1159	99.8%	1323	82.8%	1393	52.6%
Is pregnant		1		26		66
Rural residence in adulthood	1258	73.1%	1323	33.6%		-
General life satisfaction b	1244	19 [17, 21]	1323	18 [17, 20]	1386	17 [14, 20]
***Health outcomes***						
Body Mass Index (kg/m^2^)	1022	28.1±5.0	1290	25.0±4.6	1393	-
Psychological distress c	1257	3 [1,6]	1323	2 [0,4]	1393	6 [3,10]
Subjective Happiness Scale	1244	4 [4,5]	1323	3.5 [3.2, 4.0]	1392	4 [3,5]

All values displayed as mean ± standard deviation or median [25^th^ percentile, 75^th^ percentile] for continuous variables and percentage (%) for categorical variables;

a Wealth in childhood and adulthood were based on cross-sectional asset indices;

b General life satisfaction was measured using the NIH Toolbox Item Bank v2.0

c WHO SRQ-20 is a 20-item psychological distress scale. Values greater than or equal to 7 may indicate mental distress.

The distribution of SSS (Supplementary Fig. 2A–C) did not differ by sex, residence (urban vs rural in Guatemala and Philippines), or skin color (Black vs other in South Africa). The SSS measures (Supplementary Fig. 3A–C) were positively correlated with each other, while SRQ-20 and Subjective Happiness Scale (SHS) were negatively correlated (Supplementary Fig. 3D–F**)**.

### 3.1 Association of subjective social status with BMI, distress and wellbeing

Association of subjective social status with BMI, distress and wellbeing. We present associations from linear regression in (Table 2 and Fig. 2). In the model without covariate adjustment (Model 1), the respect ladder was associated with BMI in Philippines (0.15 kg/m^2^ per 1-point difference, 95% CI: 0.04, 0.25), while the economic ladder was associated with BMI in Philippines (0.31, 95%CI: 0.14, 0.49) and Guatemala (0.17, 95%CI: 0.04, 0.31). After adjusting for life course SEP and life satisfaction (Model 2), neither respect ladder, nor economic ladder were associated with BMI in Philippines. The association of BMI and economic ladder attenuated (0.07 kg/m^2^ per 1-point difference, 95%CI: -0.07, 0.20) on adjusting for all covariates (Model 3).

**Table 2:** Association of subjective social status with health and wellbeing after progressive adjustment for covariates.

	Guatemala (INCAP) *N* = 1258			Philippines (CLHNS) *N* = 1323			South Africa (Birth to Twenty plus) *N* = 1393		
	Model 1	Model 2	Model 3	Model 1	Model 2	Model 3	Model 1	Model 2	Model 3
*Perceived Community Respect*									
Body Mass Index (kg/m^2^)	0.03 (-0.07, 0.13)	0.00 (-0.1, 0.1)	0.02 (-0.07, 0.12)	0.15 (0.04, 0.25)	0.05 (-0.06, 0.15)	0.05 (-0.06, 0.15)			
SRQ-20	-0.07 (-0.15, 0)	0.00 (-0.07, 0.07)	0.02 (-0.05, 0.08)	-0.01 (-0.09, 0.07)	0.04 (-0.03, 0.12)	-0.02 (-0.1, 0.06)	-0.08 (-0.2, 0.05)	0.03 (-0.09, 0.15)	0.01 (-0.1, 0.13)
Subjective Happiness Scale	0.05 (0.03, 0.07)	0.03 (0.01, 0.04)	0.03 (0.01, 0.04)	0.06 (0.04, 0.08)	0.04 (0.03, 0.06)	0.03 (0.02, 0.05)	0.1 (0.07, 0.12)	0.07 (0.04, 0.09)	0.07 (0.04, 0.09)
*Perceived Economic Status*									
Body Mass Index (kg/m^2^)	0.17 (0.04, 0.3)	0.10 (-0.04, 0.23)	0.07 (-0.07, 0.2)	0.31 (0.14, 0.49)	0.00 (-0.19, 0.19)	-0.03 (-0.22, 0.17)			
SRQ-20	-0.1 (-0.18, -0.02)	-0.02 (-0.11, 0.06)	-0.05 (-0.13, 0.03)	-0.12 (-0.23, 0)	-0.04 (-0.17, 0.08)	-0.16 (-0.41, 0.1)	-0.27 (-0.42, -0.13)	-0.04 (-0.18, 0.1)	-0.05 (-0.2, 0.09)
Subjective Happiness Scale	0.05 (0.03, 0.07)	0.02 (0, 0.04)	0.02 (0, 0.04)	0.1 (0.07, 0.12)	0.06 (0.04, 0.08)	0.04 (0.02, 0.07)	0.13 (0.09, 0.16)	0.07 (0.04, 0.1)	0.07 (0.04, 0.1)

All coefficients displayed are for subjective social status. Model 1: Subjective social status; Model 2: Model 1 + life course SEP (wealth, maternal and own schooling, own formal employment) + life satisfaction; Model 3: Model 2 + early life covariates + adult covariates.

Crude inverse associations between the economic ladder and psychological distress also did not persist after adjustment for life course SEP, life satisfaction and other covariates (Model 3) in Philippines (-0.16 units SRQ-20 per 1-point difference in ladder, 95%CI: -0.43, 0.11) and South Africa (-0.05 units SRQ-20, 95%CI: -0.20, 0.09). The respect ladder was also not associated with psychological distress in any cohort.

Both the respect ladder (Guatemala: 0.03 [0.01, 0.04]; Philippines: 0.03 [0.02, 0.05]; South Africa: 0.07 [0.04, 0.09]) and economic ladder (Guatemala: 0.02 [0.00, 0.04]; Philippines: 0.04 [0.02, 0.07], South Africa: 0.07 [0.04, 0.10]) were positively associated with the subjective happiness scale for all three cohorts after adjusting for life course objective SEP, early and adult covariates and life satisfaction. The associations between both ladders and subjective happiness were similar in magnitude. For example, in South Africa, one-unit difference in respect ladder was associated with 0.07 unit (95%CI: 0.04, 0.09) change in the subjective happiness scale while one-unit difference in economic ladder was associated with 0.07 unit (95%CI: 0.04, 0.10) change in subjective happiness scale.

### 3.2 Effect modification of association of subjective social status with BMI, distress and wellbeing

We observed evidence suggestive of differences (Supplementary Table 2A) by sex such that the magnitude of association of the economic ladder with BMI in Guatemala (male-female difference: 0.22 kg/m^2^ per 1-point change, 95%CI: -0.06, 0.50) and Philippines (male-female difference: 0.52 kg/m^2^, 95%CI: 0.15, 0.90) was higher among male participants. We also observed potential differences by sex in Guatemala for association of the economic ladder with happiness (male – female difference: -0.05 units per 1-point change, 95%CI: -0.09, 0.00) and psychological distress (male – female difference: 0.23 units, 95%CI: 0.07, 0.39). We did not observe any other heterogeneous associations by sex, attained schooling or adult wealth (Supplementary Table 2A–C) for either ladder across cohorts since differences were small in magnitude.

### 3.3 Sensitivity analysis

The observed associations of respect and economic ladders with outcomes did not differ from the main analysis after adjusting for additional life course wealth measures (Supplementary Fig. 4). Similarly, the results did not differ from the main analysis after accounting for attrition using inverse probability weights except for association of economic status with psychological distress (Supplementary Fig. 5).

Our analysis for unmeasured confounding using e-values (Table 3) suggested that an unmeasured confounder stronger (under an assumption of equal scale) than cross-sectional wealth or formal employment would be required to nullify the observed association of the respect ladder and economic ladder with happiness across all sites. For example, the estimates of the association of wealth, and formal employment with happiness were 0.05 units (per 1 unit of wealth, 95%CI: -0.01, 0.10) and 0.06 units (95%CI: -0.03, 0.16) respectively. The e-value for the same association is 0.16 (CI: 0.01).

**Table 3:** E-values for unmeasured confounding for association of subjective social status with health and wellbeing.

	Guatemala (INCAP) N = 1258			Philippines (CLHNS) N = 1323			South Africa (Birth to Twenty plus) N = 1393		
	Coefficient for Wealth in adulthood	Coefficient for Formal employment	e-value	Coefficient for Wealth in adulthood	Coefficient for Formal employment	e-value	Coefficient for Wealth in adulthood	Coefficient for Formal employment	e-value
*Perceived Community Respect*									
Body Mass Index (kg/m^2^)	0.58 (0.17, 0.99)	-0.13 (-0.78, 0.53)	0.07; CI: 0	0.68 (0.34, 1.01)	0.4 (-0.11, 0.9)	0.11; CI: 0			
SRQ-20	-0.26 (-0.51, -0.02)	0.07 (-0.36, 0.50)	0.08; CI: 0	0.05 (-0.18, 0.28)	-0.38 (-0.83, 0.06)	0.09; CI: 0	-0.08 (-0.34, 0.19)	0.71 (0.22, 1.2)	0.05; CI: 0
Subjective Happiness Scale	0.05 (-0.01, 0.1)	0.06 (-0.03, 0.16)	0.2; CI: 0.11	0.02 (-0.02, 0.06)	0.05 (-0.01, 0.1)	0.26; CI: 0.21	0 (-0.06, 0.06)	-0.11 (-0.21, -0.01)	0.31; CI: 0.23
*Perceived Economic Status*									
Body Mass Index (kg/m^2^)	0.55 (0.14, 0.96)	-0.12 (-0.77, 0.54)	0.13; CI: 0	0.71 (0.36, 1.06)	0.41 (-0.1, 0.92)	0.09; CI: 0			
SRQ-20	-0.23 (-0.48, 0.01)	0.07 (-0.36, 0.51)	0.13; CI: 0	0.17 (-0.14, 0.48)	-0.31 (-1, 0.38)	0.26; CI: 0	-0.07 (-0.33, 0.19)	0.73 (0.24, 1.22)	0.11; CI: 0
Subjective Happiness Scale	0.04 (-0.02, 0.1)	0.07 (-0.02, 0.17)	0.16; CI: 0.01	0.01 (-0.03, 0.05)	0.04 (-0.02, 0.1)	0.3; CI: 0.21	-0.01 (-0.07, 0.05)	-0.11 (-0.21, -0.01)	0.31; CI: 0.23

Coefficients displayed are for association of wealth in adulthood and formal employment with different health outcomes based on Model 3 (adjusted for life course socio-economic position, early life and adult life covariates, life satisfaction).

## 4 Discussion

Our results from birth cohorts in three LMICs suggest that subjective social status, as either community respect or economic status, is positively associated with happiness but is not associated with weight status (participants were predominantly overweight or obese), and psychological distress. These associations did not differ by levels of schooling or wealth, although there were some differences by sex. The results were robust to alternate model specifications, and suggested only an unmeasured confounder stronger than adult wealth and formal employment, reliable markers of SEP in LMICs, could nullify the observed association.

This research extends previous investigation of subjective social status with outcomes in Guatemala by including two new settings and additionally considering life satisfaction, marital status, and whether they had children (Varghese et al., 2021). Subjective wellbeing consists of three domains: evaluative wellbeing (life satisfaction), emotional wellbeing (experienced wellbeing) and eudaimonic wellbeing (e.g. meaning and purpose). The first is a long-term evaluation of one's life experiences, while the second is related to one's frequency of positive (positive affect; like happiness and pleasure) and negative (negative affect; like shame, fear and anger) feelings (National Research Council, 2013). Subjective social status and happiness (an indicator of positive affect) were positively associated in all three cohorts, after adjusting for markers of objective SEP, consistent with research from representative samples of adults in 29 countries, including Philippines and South Africa (Prag et al., 2016). Low SSS was previously shown to be associated with chronic negative affect, potentially as lack of power (control) and social acceptance in western societies (Kraus et al., 2013; Jackson et al., 2011; Anderson et al., 2012). A randomized study among three hundred adult participants from the US suggested that low subjective status was associated with negative affect (Kraus et al., 2013).

Results from a meta-analysis of 38 studies (6 were from LMICs) and from 20 household surveys in 18 countries (11 were LMICs) suggest higher subjective social status was associated with better mental health (Zell et al., 2018; Scott et al., 2014). Additionally, the observed null findings for association of cross-sectional wealth and psychological distress after adjusting for subjective social status is consistent with results from LMICs such as Myanmar and Uganda (Sasaki, Shobugawa, and Nozaki, 2021; Smith et al., 2019). The null findings for subjective social status and BMI in Philippines and Guatemala were also consistent with research from East Asia and Mexico, as well as Adler et.al.’s original study among women in USA (Adler et al., 2000; Fernald, 2007; Frerichs et al., 2014). However, other research from high-income countries (England, USA) has shown negative associations of subjective status with BMI (Tang et al., 2016; Dhurandhar et al., 2018). Together with these results, our findings suggest that subjective social status may be a reliable marker for wellbeing but not weight status in non-western societies. Our analysis for assessing robustness of these associations compared it to the association of employment and wealth with BMI, SRQ-20 and wellbeing. A confounder that is stronger than wealth and employment, for example, personality traits such as self-esteem or history of mental health issues such as depression may nullify the observed association with wellbeing (Hoebel and Lampert, 2020).

Previous research from a nationally-representative predominantly rural and food insecure population in Malawi asked the economic ladder question relative to an unspecified frame of reference (Ravallion and Lokshin, 2010). The ladder was compared to cross-sectional asset indices (relative wealth) for identifying at-risk households to improve targeting of health and other interventions based on US$1-a-day poverty (Howe et al., 2011). In this population, the ladder measure was more strongly correlated with absolute poverty, as measured by household expenditures, than the asset index, since the latter was partly determined by community infrastructure (such as electricity) (Howe et al., 2011). The asset index may therefore incorrectly classify the poverty status of households. Though both the ladder and the asset index measured relative position, the former may be more useful for targeting health interventions at the population-level especially when relative wealth categories (such as quintiles) may not be easily translatable into absolute poverty. This could be the case with skewed distributions (high inequality), acute financial distress (such as famine or natural disasters) and studies in catchment areas with high rates of poverty. However, the subjective nature of the measure deems it susceptible to measurement error and therefore, a combination of many SEP measures could be used for identifying at-risk households.

### 4.1 Strengths and limitations

Our study has many strengths, including duration of follow-up, availability of data on early life SEP, adjustment for life course SEP and life satisfaction as well as outcome data collected using consistent methodology across three cohorts. However, there are some limitations. Firstly, our outcomes and adult SEP were measured cross-sectionally. Hence, our results that suggest SSS may predict health (social causation) is susceptible to reverse causality such that poor health may result in lower SSS (health selection). However, evidence from Europe suggests that both social causation and health selection operate in early life and adolescence, while social causation is the predominant mechanism in adulthood (Hoffmann et al., 2018). Secondly, the measures of subjective social status, SRQ-20 and Subjective Happiness Scale were not validated in the context of these particular countries. These measures have been used previously in South Africa as well as other low- and middle-income countries (Brazil, India, Indonesia, Myanmar, Uganda, Vietnam, and Zambia) (Sasaki et al., 2021; Prag et al., 2016; Smith et al., 2019; Camelo Ldo et al., 2014; Nobles et al., 2013; Hamad et al., 2008; Cole, 2012; de Quadros Lde et al., 2015; Extremera and Fernández-Berrocal, 2014; Giang et al., 2006; Patel et al., 2008; van der Westhuizen et al., 2016). Third, the birth cohorts in our study were not designed to be nationally representative. All the cohorts were community-based and we did not observe differential loss to follow-up. We also did not observe any differences in distribution of SSS by sex and region of residence. Using only a single item from the Subjective Happiness Scale for South Africa may bias our reported estimates, compared to the other cohorts. However, given the consistent associations observed between SSS and different outcomes, we believe our results are generalizable across settings in spite of these limitations. Fourth, we did not explore the heterogeneity of association with high school completion or with different types of non-formal engagement (informal, unemployed, not seeking work) in the job market since these are important indicators of objective SEP in LMIC settings. Furthermore, we did not estimate three-way heterogeneity with schooling or wealth by sex due to low sample sizes. Finally, though our hypotheses were decided a priori, we test the association of two measures of SSS with three outcomes across many model formulations, potentially warranting an adjustment of significance level for multiple comparisons. Such an analysis was beyond the scope of this paper and hence was not included.

## 5 Conclusion

Our research demonstrates consistent associations for SSS and happiness, beyond general life satisfaction and objective SEP, across three cohorts from LMICs at different stages of economic development. Further research on the implications of low subjective status for individual wellbeing in LMIC contexts ought to be conducted given that there might be cultural differences in how subjective status manifested, and how status comparisons may be influenced by cultural or community norms as well as life course SEP (Park et al., 2013).

## Ethics approval and consent to participate

All participants gave written informed consent before participation. Ethical approval for the latest study wave for the cohorts were obtained from the Institutional Review Board of Emory University, USA (Protocol 95960), Institute of Nutrition for Central America and Panama, Guatemala (Protocol CIE-REV-072-2017), Research Ethics Committee at University of San Carlos, Philippines (Protocol 006/2018-01-borja), and Human Research Ethics Committee at University of Witswatersrand, South Africa (Certificate No. M180225).

## Funding

Funded by the Bill and Melinda Gates Foundation [grant number OPP1164115] for data collection in Guatemala, Philippines, and South Africa for data management and analysis. The Birth to Twenty Plus Cohort is supported by the South African Medical Research Council, DSI-NRF Centre of Excellence in Human Development at the University of the Witwatersrand, Johannesburg, South Africa, and the Wellcome Trust [grant numbers 077210/Z/05/Z, 092097/Z/10/Z].

## Data availability statement

The code for the analysis is available on https://github.com/jvargh7/cohorts-subjective-status. Data will be available upon reasonable request addressed to the principal investigators at each study site.

## Consent for publication

Not applicable

## CRediT authorship contribution statement

**Jithin Sam Varghese:** Conceptualization, Methodology, Formal analysis, Writing – original draft. **Rachel Waford Hall:** Methodology, Writing – review & editing. **Linda S Adair:** Methodology, Data curation, Writing – review & editing. **Shivani A Patel:** Methodology, Writing – review & editing. **Reynaldo Martorell:** Methodology, Data curation, Writing – review & editing. **Delia E. Belleza:** Methodology, Data curation, Writing – review & editing. **Maria F Kroker-Lobos:** Methodology, Data curation, Project administration, Writing – review & editing. **Nanette R. Lee:** Methodology, Data curation, Project administration, Writing – review & editing. **Lukhanyo H. Nyati:** Methodology, Data curation, Project administration, Writing – review & editing. **Manuel Ramirez-Zea:** Methodology, Data curation, Project administration, Writing – review & editing. **Linda M Richter:** Methodology, Data curation, Project administration, Writing – review & editing. **Aryeh D. Stein:** Conceptualization, Methodology, Funding acquisition, Supervision, Writing – original draft, Writing – review & editing.

## Declaration of Competing Interest

The authors declare that they have no known competing financial interests or personal relationships that could have appeared to influence the work reported in this paper.

## Appendix Supplementary materials

Image, application 1
