# Supplementary material for: Subjective social status is associated with happiness but not weight status or psychological distress: An analysis of three prospective birth cohorts from low- and middle-income countries
Source: Wellbeing Space Soc. 2022;3:None. doi: 10.1016/j.wss.2022.100115 (PMC9732742; doi:10.1016/j.wss.2022.100115)
Supplement: Supplementary file 1 [file mmc1.docx]

**Table of Contents**

[Supplementary Note 1 Details on Sensitivity Analyses for robustness of findings 2](#_Toc87618440)

[Supplementary Fig 1 Flowchart for analytic sample construction in three low- and middle-income country cohorts 4](#_Toc87618441)

[Supplementary Fig 2A Distribution of subjective social status in Guatemala 5](#_Toc87618442)

[Supplementary Fig 2B. Distribution of subjective social status in Philippines 6](#_Toc87618443)

[Supplementary Fig 2C Distribution of subjective social status in South Africa 7](#_Toc87618444)

[Supplementary Fig 3. Bivariate association of self-reported measures in three LMIC birth cohorts 8](#_Toc87618445)

[Supplementary Fig 4. Linear regression after adjusting for life course wealth measures for 3 cohorts x 3 outcomes x 2 SSS measures 9](#_Toc87618446)

[Supplementary Fig 5. Linear regression after inverse probability censoring weights for 3 cohorts x 3 outcomes x 2 SSS measures 10](#_Toc87618447)

[Supplementary Table 1 Baseline characteristics by participation status in adulthood 11](#_Toc87618448)

[Supplementary Table 2A Regression coefficients for effect modification by sex 12](#_Toc87618449)

[Supplementary Table 2B Regression coefficients for effect modification by schooling 14](#_Toc87618450)

[Supplementary Table 2C Regression coefficients for effect modification by wealth in adulthood 16](#_Toc87618451)

# Supplementary Note 1 Details on Sensitivity Analyses for robustness of findings

***Analysis 1. Residual confounding by life course relative wealth***

Model 3:

E[Outcome] = b0 + b1 Subjective Social Status + c X + d Z

where X: Early life SEP (maternal schooling, early life relative wealth) and adult SEP at time of outcome measurement (attained schooling, formal employment, adult relative wealth)

where Z: early life and adult covariates (Section 2.4 in Manuscript)

Analysis 1 Model: b0 + b1 Subjective Social Status + c X + d Z + e W

where W: relative wealth in other study waves measured prior to subjective social status ladders.

- Guatemala: in 1987, 1996, 2002
- Philippines: in 1991, 1994, 1998, 2002, 2005, 2009
- South Africa: in 1997, 2002, 2006, 2012

***Analysis 2. Extent of unmeasured confounding for SSS-Outcome association***

Below figure is reproduced from Varghese 2021 SSMPH (Supplementary Figure 2). “e” is the e-value for unmeasured confounding or the minimum strength of association an unmeasured confounder should have with both the exposure (subjective social status) and outcome (such as BMI, psychological distress using WHO Self-Reported Questionnaire-20 and happiness using Subjective Happiness Scale) to nullify the observed association (β).


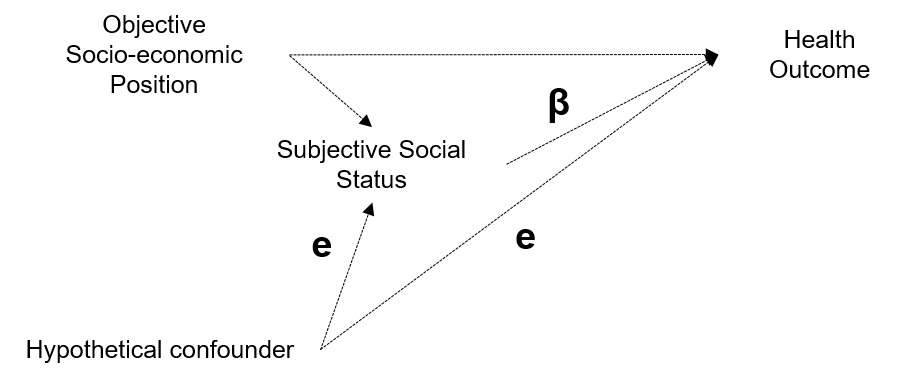


***Analysis 3. Censoring weights for non-participation due to death or non-response***

W_death_ : Weight for death: 1/Pr[Alive = 1|Early life covariates]

Wsss: Weight for providing SSS: 1/Pr[Provided SSS = 1|Early life covariates]

W_Outcome_: Weight for providing each health outcome: 1/Pr[Provided Outcome = 1|Early life covariates, Adult covariates]

Censoring weight = W_death_ * Wsss * W_Outcome_

# Supplementary Fig 1 Flowchart for analytic sample construction in three low- and middle-income country cohorts


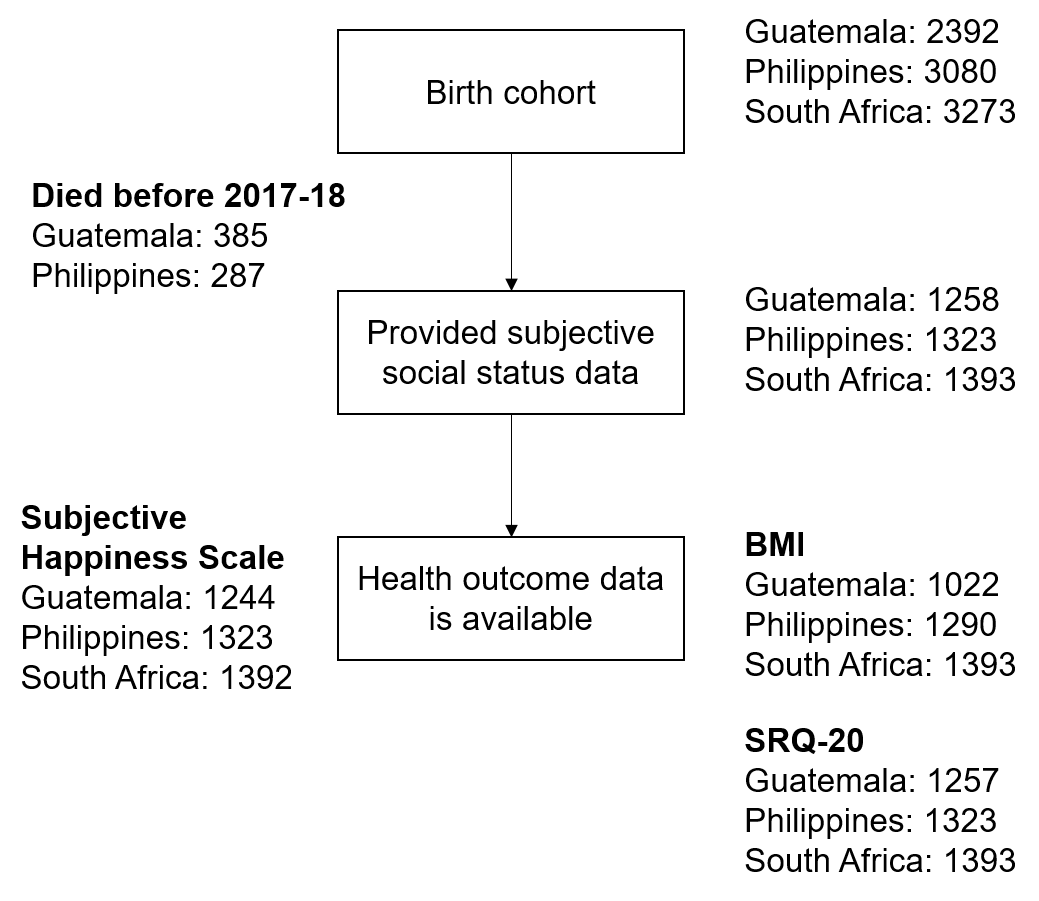


# Supplementary Fig 2A Distribution of subjective social status in Guatemala


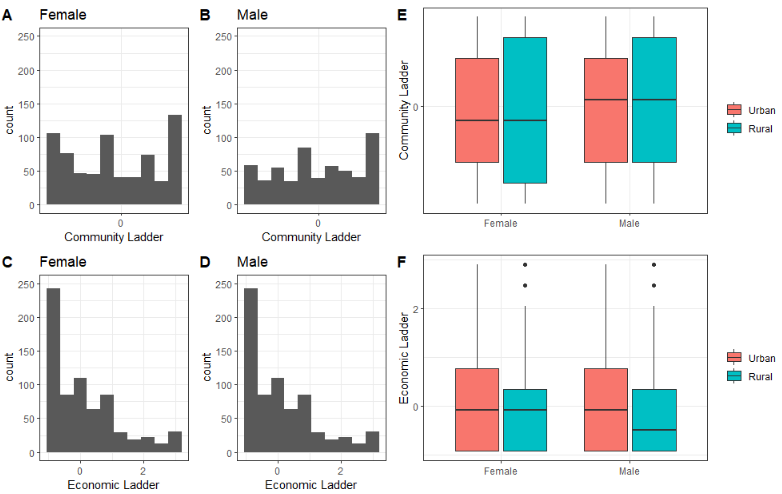


# Supplementary Fig 2B. Distribution of subjective social status in Philippines


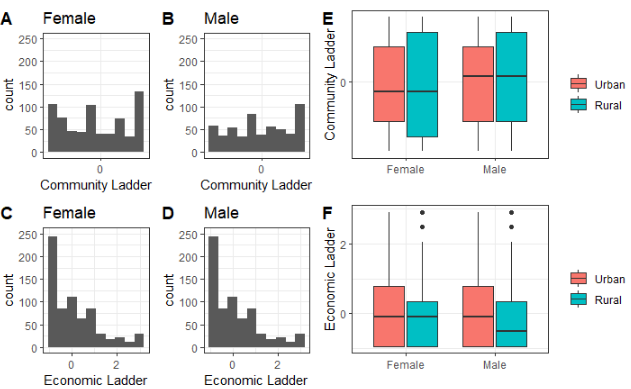


# Supplementary Fig 2C Distribution of subjective social status in South Africa


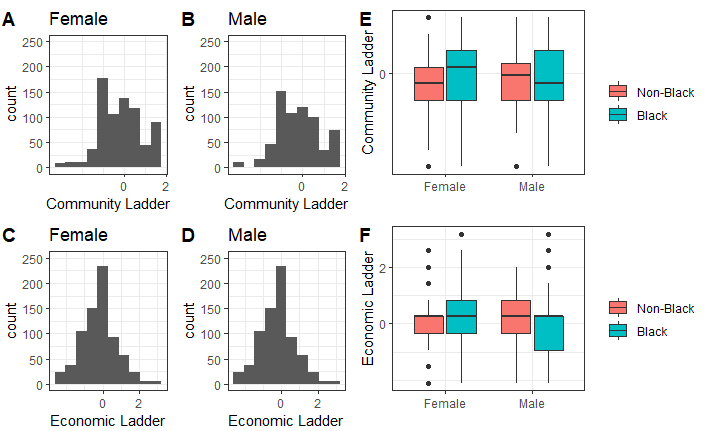


# Supplementary Fig 3. Bivariate association of self-reported measures in three LMIC birth cohorts


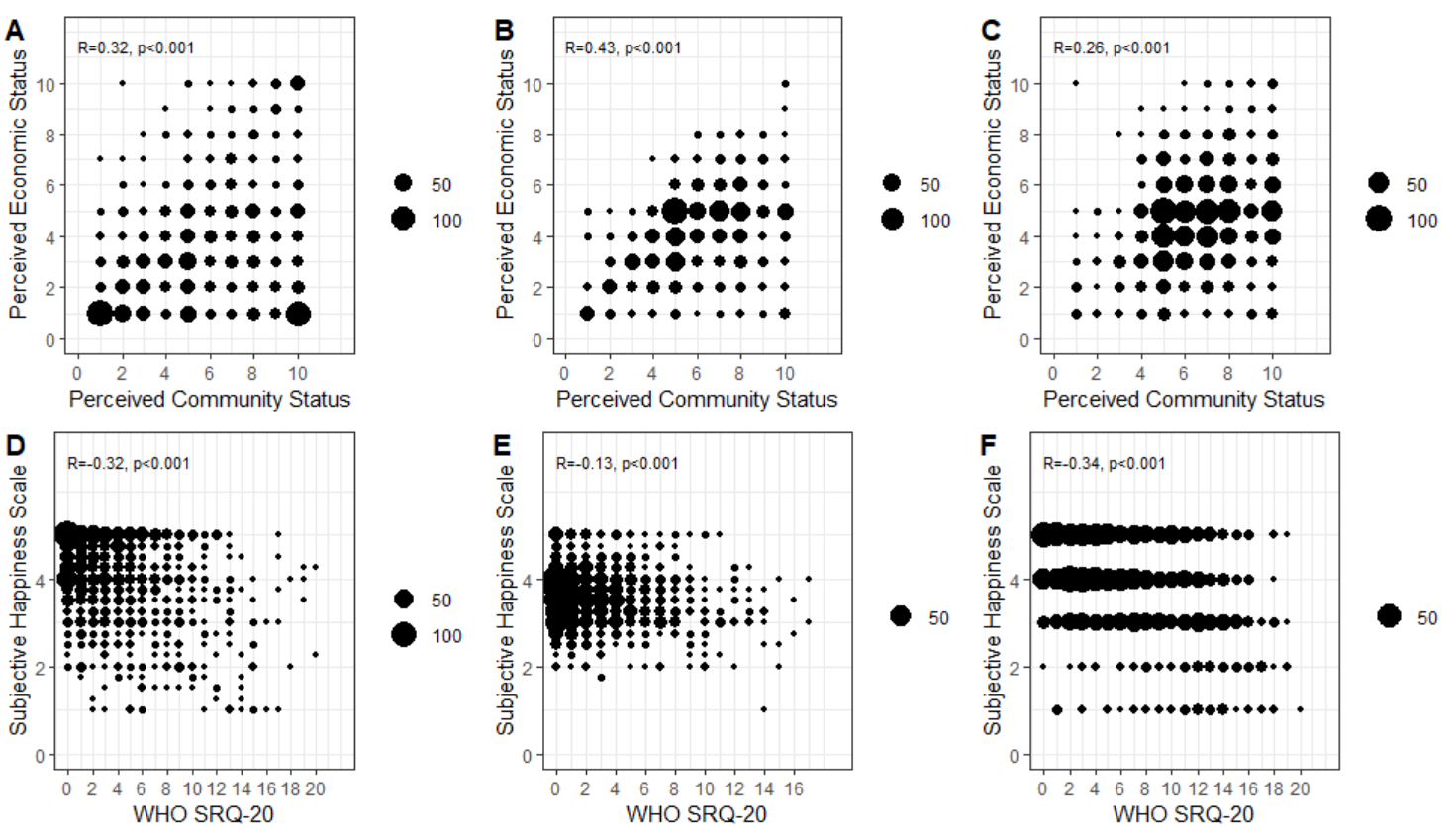


Panels A-C are for Guatemala, Philippines and South Africa respectively. Panels D-F are for Guatemala, Philippines and South Africa respectively.

# Supplementary Fig 4. Linear regression after adjusting for life course wealth measures for 3 cohorts x 3 outcomes x 2 SSS measures


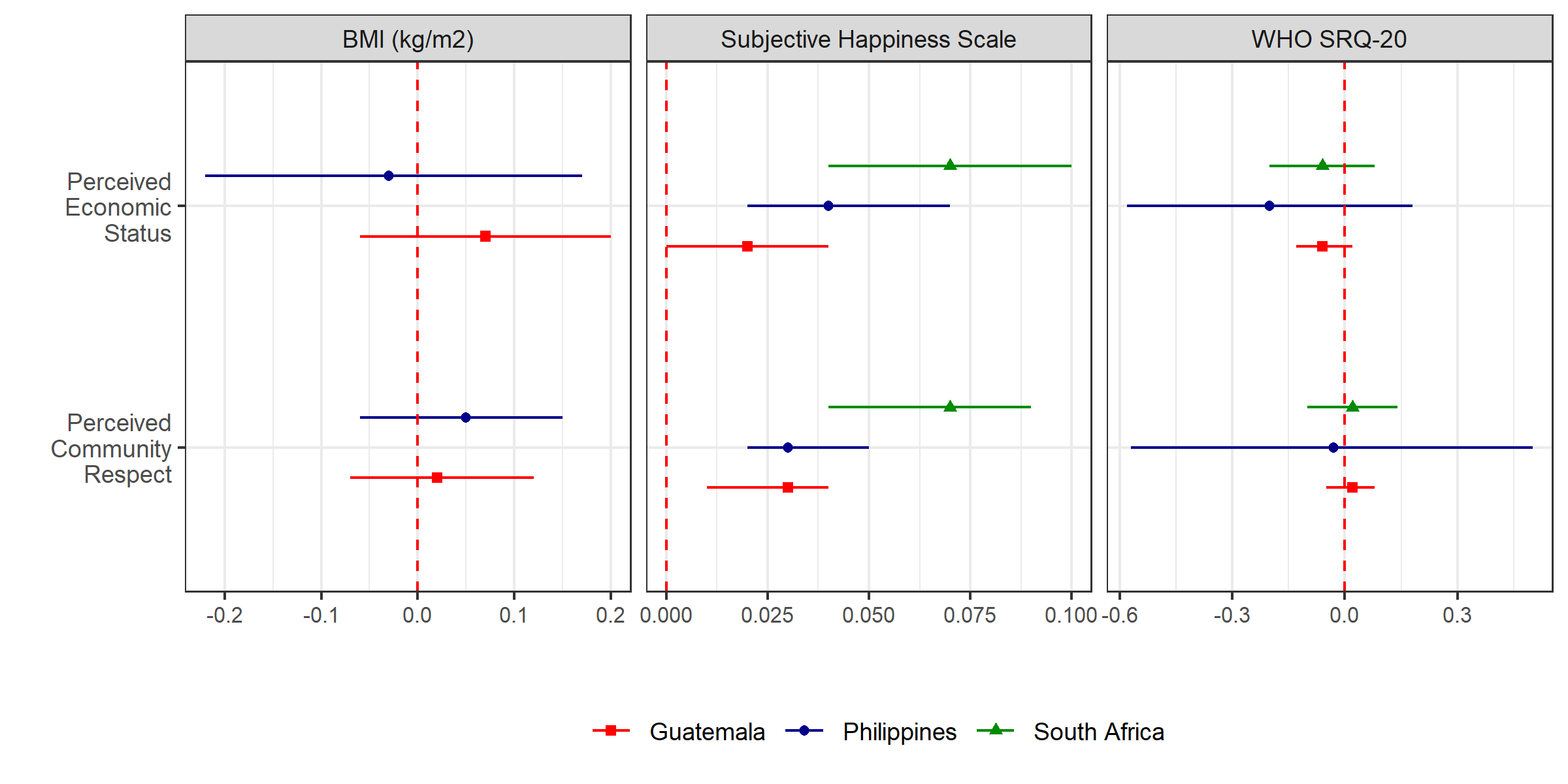


# Supplementary Fig 5. Linear regression after inverse probability censoring weights for 3 cohorts x 3 outcomes x 2 SSS measures


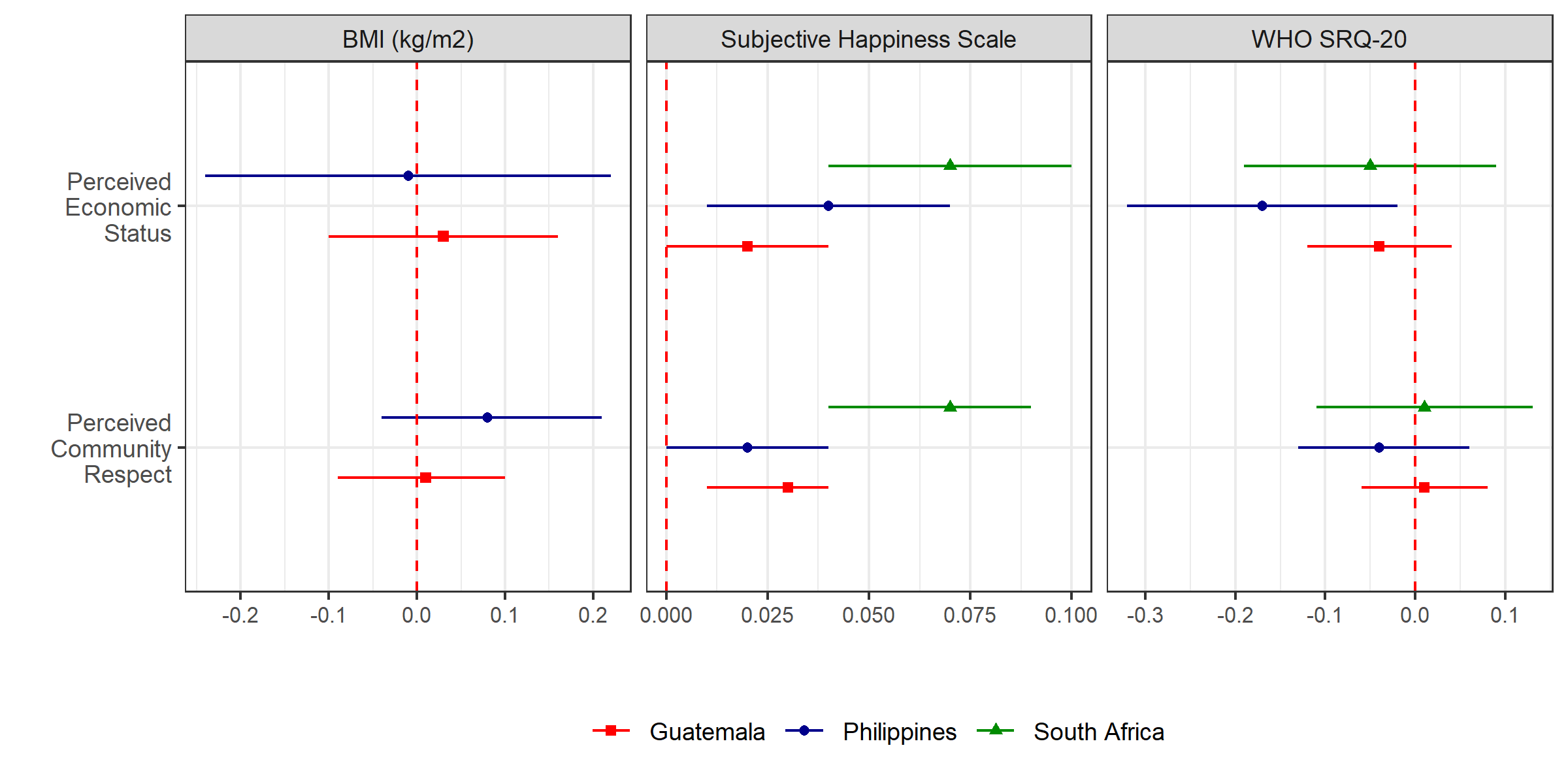


# Supplementary Table 1 Baseline characteristics by participation status in adulthood

|  | **Guatemala** | | | **Philippines** | | | **South Africa** | |
| --- | --- | --- | --- | --- | --- | --- | --- | --- |
|  | **Died** | **Did not respond** | **Participated** | **Died** | **Did not respond** | **Participated** | **Did not respond or Died** | **Participated** |
| **N** | **385** | **749** | **1258** | **287** | **1470** | **1323** | **1880** | **1393** |
| Maternal schooling | 0 [0, 2] | 1 [0, 2] | 1 [0, 2] | 6 [4, 7] | 7 [6, 10] | 6 [5, 9] | 9 [9, 11.5] | 9 [9, 11.5] |
| Maternal age | 27.5±7.7 | 26.8±7.3 | 27.0±7.1 | 26.4±6.6 | 26.0±5.8 | 26.5±6.1 | 26.1±5.9 | 25.8±6.3 |
| Wealth in childhood | -0.1±0.9 | <0.1±0.8 | -0.1±0.9 | -0.3±0.8 | 0.1±1.1 | -0.1±0.9 | 0.1±1.0 | <0.1±0.8 |
| Male | 58.4% | 59.3% | 44.6% | 61.8% | 50.5% | 53.9% | 49.5% | 47.4% |
| Birth order | 4 [2, 4] | 4 [2, 4] | 4 [2, 4] | 3 [2, 4] | 3 [2, 4] | 3 [2, 4] | 2 [1, 3] | 2 [1, 3] |
| Atole supplementation | 55.6% | 52.6% | 52.5% |  |  |  |  |  |
| Rural residence |  |  |  | 26.8.1% | 18.6% | 28.0% |  |  |
| Black |  |  |  |  |  |  | 71.1% | 88.4% |

# Supplementary Table 2A Regression coefficients for effect modification by sex

|  |  | **Guatemala (INCAP)**  **N = 1258** | | **Philippines (CLHNS)**  **N = 1323** | | **South Africa (Birth to Twenty plus)**  **N = 1393** | |
| --- | --- | --- | --- | --- | --- | --- | --- |
| **Effect modification by sex** | **Contrast** | **Coefficient** | **LRT result** | **Coefficient** | **LRT result** | **Coefficient** | **LRT result** |
| ***Perceived Community Respect*** |  |  |  |  |  |  |  |
| Body Mass Index (kg/m^2^) | Female | -0.04  (-0.17, 0.10) | 45.78, p = 0.011 | -0.03  (-0.26, 0.20) | 22.21, p = 0 |  |  |
|  | Male | 0.08  (-0.07, 0.22) |  | 0.09  (-0.04, 0.22) |  |  |  |
|  | Difference | 0.11  (-0.09, 0.32) |  | 0.13  (-0.14, 0.39) |  |  |  |
| Subjective Happiness Scale | Female | 0.03  (0.01, 0.05) | 0.33, p = 0.574 | 0.04  (0.01, 0.07) | 0.14, p = 0.713 | 0.05  (0.02, 0.09) | 1.2, p = 0.273 |
|  | Male | 0.02  (-0.00, 0.04) |  | 0.03  (0.01, 0.04) |  | 0.08  (0.04, 0.12) |  |
|  | Difference | -0.01  (-0.04, 0.02) |  | -0.01  (-0.04, 0.02) |  | 0.03  (-0.03, 0.08) |  |
| SRQ-20 | Female | 0.00  (-0.09, 0.10) | 6.07, p = 0.016 | -0.01  (-0.15, 0.14) | 2.24, p = 0.134 | -0.04  (-0.21, 0.12) | 1.08, p = 0.299 |
|  | Male | 0.05  (-0.03, 0.13) |  | -0.04  (-0.13, 0.06) |  | 0.08  (-0.09, 0.24) |  |
|  | Difference | 0.05  (-0.08, 0.17) |  | -0.03  (-0.20, 0.14) |  | 0.12  (-0.11, 0.35) |  |
| ***Perceived Economic Status*** |  |  |  |  |  |  |  |
| Body Mass Index (kg/m^2^) | Female | -0.03  (-0.20, 0.13) | 78.86, p = 0 | -0.35  (-0.67, -0.02) | 189.46, p = 0 |  |  |
|  | Male | 0.19  (-0.04, 0.41) |  | 0.18  (-0.05, 0.40) |  |  |  |
|  | Difference | 0.22  (-0.06, 0.50) |  | 0.52  (0.15, 0.90) |  |  |  |
| Subjective Happiness Scale | Female | 0.04  (0.01, 0.06) | 3.21, p = 0.074 | 0.04  (-0.00, 0.08) | 0.02, p = 0.875 | 0.10  (0.05, 0.14) | 2.85, p = 0.091 |
|  | Male | -0.01  (-0.04, 0.03) |  | 0.05  (0.02, 0.07) |  | 0.05  (-0.00, 0.10) |  |
|  | Difference | -0.05  (-0.09, -0.00) |  | 0.01  (-0.04, 0.05) |  | -0.05  (-0.11, 0.02) |  |
| SRQ-20 | Female | -0.13  (-0.23, -0.02) | 83.01, p = 0 | -0.28  (-0.50, -0.06) | -5540.81, p = 1 | -0.13  (-0.34, 0.07) | 1.32, p = 0.25 |
|  | Male | 0.10  (-0.01, 0.22) |  | -0.10  (-0.25, 0.05) |  | 0.02  (-0.16, 0.20) |  |
|  | Difference | 0.23  (0.07, 0.39) |  | 0.18  (-0.07, 0.43) |  | 0.15  (-0.11, 0.42) |  |

# Supplementary Table 2B Regression coefficients for effect modification by schooling

|  |  | **Guatemala (INCAP)**  **N = 1258** | | **Philippines (CLHNS)**  **N = 1323** | | **South Africa (Birth to Twenty plus)**  **N = 1393** | |
| --- | --- | --- | --- | --- | --- | --- | --- |
| **Effect modification by schooling** | **Contrast** | **Coefficient** | **LRT result** | **Coefficient** | **LRT result** | **Coefficient** | **LRT result** |
| ***Perceived Community Respect*** |  |  |  |  |  |  |  |
| Body Mass Index (kg/m^2^) | SSS at mean years | 0.01  (-0.09, 0.11) | 4.05, p = 0.451 | 0.06  (-0.07, 0.18) | 11.05, p = 0.001 |  |  |
|  | SSS at mean years + 1 y | 0.02  (-0.09, 0.13) |  | 0.07  (-0.07, 0.21) |  |  |  |
|  | Difference | 0.01  (-0.02, 0.03) |  | 0.01  (-0.02, 0.04) |  |  |  |
| Subjective Happiness Scale | SSS at mean years | 0.03  (0.01, 0.04) | 0.53, p = 0.468 | 0.03  (0.02, 0.04) | 0.14, p = 0.708 | 0.07  (0.04, 0.09) | 0.09, p = 0.771 |
|  | SSS at mean years + 1 y | 0.02  (0.01, 0.04) |  | 0.03  (0.01, 0.04) |  | 0.07  (0.04, 0.10) |  |
|  | Difference | -0.00  (-0.01, 0.00) |  | -0.00  (-0.01, 0.00) |  | 0.00  (-0.02, 0.02) |  |
| SRQ-20 | SSS at mean years | 0.03  (-0.03, 0.09) | 34.38, p = 0 | -0.03  (-0.11, 0.06) | -20.45, p = 1 | 0.00  (-0.12, 0.12) | 2.27, p = 0.132 |
|  | SSS at mean years + 1 y | 0.05  (-0.02, 0.11) |  | -0.03  (-0.11, 0.06) |  | -0.05  (-0.20, 0.10) |  |
|  | Difference | 0.02  (-0.00, 0.03) |  | -0.00  (-0.02, 0.02) |  | -0.05  (-0.12, 0.02) |  |
| ***Perceived Economic Status*** |  |  |  |  |  |  |  |
| Body Mass Index  (kg/m^2^) | SSS at mean years | 0.05  (-0.09, 0.18) | 5.9, p = 0.411 | -0.04  (-0.24, 0.17) | 26.74, p = 0 |  |  |
|  | SSS at mean years + 1 y | 0.05  (-0.09, 0.20) |  | -0.06  (-0.29, 0.16) |  |  |  |
|  | Difference | 0.01  (-0.03, 0.04) |  | -0.03  (-0.08, 0.02) |  |  |  |
| Subjective Happiness Scale | SSS at mean years | 0.02  (-0.00, 0.04) | 3.48, p = 0.062 | 0.04  (0.02, 0.07) | 0.02, p = 0.898 | 0.07  (0.04, 0.10) | 0, p = 0.957 |
|  | SSS at mean years + 1 y | 0.01  (-0.01, 0.03) |  | 0.04  (0.02, 0.07) |  | 0.07  (0.03, 0.11) |  |
|  | Difference | -0.01  (-0.01, -0.00) |  | -0.00  (-0.01, 0.01) |  | 0.00  (-0.02, 0.02) |  |
| SRQ-20 | SSS at mean years | -0.04  (-0.12, 0.04) | 10.51, p = 0.001 | -0.18  (-0.31, -0.04) | -1411.14, p = 1 | -0.04  (-0.19, 0.10) | 1.15, p = 0.283 |
|  | SSS at mean years + 1 y | -0.03  (-0.11, 0.05) |  | -0.20  (-0.34, -0.05) |  | -0.00  (-0.17, 0.16) |  |
|  | Difference | 0.01  (-0.01, 0.03) |  | -0.02  (-0.05, 0.01) |  | 0.04  (-0.04, 0.12) |  |

# Supplementary Table 2C Regression coefficients for effect modification by wealth in adulthood

|  |  | **Guatemala**  **(INCAP)**  **N = 1258** | | **Philippines**  **(CLHNS)**  **N = 1323** | | **South Africa**  **(Birth to Twenty plus)**  **N = 1393** | |
| --- | --- | --- | --- | --- | --- | --- | --- |
| **Effect modification by wealth** | **Contrast** | **Coefficient** | **LRT result** | **Coefficient** | **LRT result** | **Coefficient** | **LRT result** |
| ***Perceived Community Respect*** |  |  |  |  |  |  |  |
| Body Mass Index  (kg/m^2^) | SSS at mean wealth | 0.01  (-0.09, 0.11) | 2.41, p = 0.396 | 0.06  (-0.07, 0.19) | 16.52, p = 0 |  |  |
|  | SSS at mean wealth + 1 unit | 0.01  (-0.14, 0.15) |  | 0.11  (-0.10, 0.32) |  |  |  |
|  | Difference | -0.00  (-0.10, 0.09) |  | 0.05  (-0.07, 0.17) |  |  |  |
| Subjective Happiness Scale | SSS at mean wealth | 0.03  (0.01, 0.04) | 0.04, p = 0.849 | 0.03  (0.02, 0.05) | 0.04, p = 0.845 | 0.07  (0.04, 0.09) | 0.34, p = 0.563 |
|  | SSS at mean wealth + 1 unit | 0.02  (0.00, 0.05) |  | 0.03  (0.01, 0.05) |  | 0.07  (0.04, 0.11) |  |
|  | Difference | -0.00  (-0.02, 0.01) |  | -0.00  (-0.02, 0.01) |  | 0.01  (-0.02, 0.04) |  |
| SRQ-20 | SSS at mean wealth | 0.04  (-0.03, 0.10) | 75.49, p = 0 | -0.02  (-0.10, 0.06) | 1.45, p = 0.229 | 0.00  (-0.11, 0.12) | 3.02, p = 0.082 |
|  | SSS at mean wealth + 1 unit | 0.12  (0.03, 0.21) |  | 0.00  (-0.12, 0.13) |  | -0.09  (-0.26, 0.08) |  |
|  | Difference | 0.08  (0.02, 0.15) |  | 0.02  (-0.06, 0.10) |  | -0.10  (-0.21, 0.01) |  |
| ***Perceived Economic Status*** |  |  |  |  |  |  |  |
| Body Mass Index  (kg/m^2^) | SSS at mean wealth | 0.04  (-0.09, 0.18) | 28.79, p = 0.1 | -0.03  (-0.23, 0.18) | 5.12, p = 0.035 |  |  |
|  | SSS at mean wealth + 1 unit | -0.01  (-0.22, 0.19) |  | -0.06  (-0.39, 0.26) |  |  |  |
|  | Difference | -0.06  (-0.20, 0.08) |  | -0.04  (-0.24, 0.17) |  |  |  |
| Subjective Happiness Scale | SSS at mean wealth | 0.02  (-0.00, 0.04) | 0, p = 0.966 | 0.04  (0.02, 0.07) | 0.06, p = 0.811 | 0.07  (0.04, 0.11) | 1.81, p = 0.179 |
|  | SSS at mean wealth + 1 unit | 0.02  (-0.01, 0.05) |  | 0.04  (0.01, 0.07) |  | 0.09  (0.05, 0.13) |  |
|  | Difference | -0.00  (-0.02, 0.02) |  | -0.00  (-0.02, 0.02) |  | 0.02  (-0.02, 0.05) |  |
| SRQ-20 | SSS at mean wealth | -0.04  (-0.12, 0.04) | 3.8, p = 0.053 | -0.17  (-0.30, -0.04) | -1148.87, p = 1 | -0.05  (-0.20, 0.09) | 0.01, p = 0.925 |
|  | SSS at mean wealth + 1 unit | -0.02  (-0.13, 0.10) |  | -0.17  (-0.35, 0.00) |  | -0.06  (-0.26, 0.14) |  |
|  | Difference | 0.03  (-0.05, 0.10) |  | -0.00  (-0.11, 0.11) |  | -0.01  (-0.14, 0.13) |  |
